# Fluoride exposure and pubertal development in children living in Mexico City

**DOI:** 10.1186/s12940-019-0465-7

**Published:** 2019-03-29

**Authors:** Yun Liu, Martha Téllez-Rojo, Howard Hu, Brisa N. Sánchez, E. Angeles Martinez-Mier, Niladri Basu, Adriana Mercado-García, Maritsa Solano-González, Karen E. Peterson

**Affiliations:** 1 Department of Nutritional Sciences, University of Michigan School of Public Health, Ann Arbor, Michigan USA; 2 Nutrition and Health Research, National Institute of Public Health, Ave. Universidad 655, Santa María Ahuacatitlán, Cuernavaca, Mor 62100 México; 3 Occupational and Environmental Health, Dalla Lana School of Public Health, University of Toronto, Toronto, Canada; 4 Department of Biostatistics, University of Michigan School of Public Health, Ann Arbor, Michigan USA; 5 Department of Cariology, Operative Dentistry and Dental Public Health, Indiana University School of Dentistry, Indianapolis, Indiana USA; 6 Faculty of Agricultural and Environmental Sciences, McGill University, Montreal, Quebec Canada

**Keywords:** Fluoride, Puberty, Pubic hair, Genitalia, Menarche

## Abstract

**Background:**

Previous animal and ecological studies have provided evidence for an earlier sexual maturation in females in relation to fluoride exposure; however, no epidemiological studies have examined the association between fluoride exposure and pubertal development in both boys and girls using individual-level biomarkers of fluoride. Capitalizing on an ongoing Mexican birth cohort study, we examined the association between concurrent urinary fluoride levels and physical markers of pubertal development in children.

**Methods:**

We conducted a cross-sectional study of 157 boys and 176 girls at age 10–17 years living in Mexico City. We used ion-selective electrode-based diffusion methods to assess fluoride levels in urine, adjusting for urinary specific gravity. Pubertal stages were evaluated by a trained physician. Associations of fluoride with pubertal stages and age at menarche were studied using ordinal regression and Cox proportional-hazard regression, respectively.

**Results:**

In the entire sample, the geometric mean and interquartile range (IQR) of urinary fluoride (specific gravity adjusted) were 0.59 mg/L and 0.31 mg/L, respectively. In boys, our analysis showed that a one-IQR increase in urinary fluoride was associated with later pubic hair growth (OR = 0.71, 95% CI: 0.51–0.98, *p* = 0.03) and genital development (OR = 0.71, 95% CI: 0.53–0.95, *p* = 0.02). No significant associations were found in girls, although the direction was negative.

**Conclusions:**

Childhood fluoride exposure, at the levels observed in our study, was associated with later pubertal development among Mexican boys at age 10–17 years. Further research is needed to confirm these findings.

**Electronic supplementary material:**

The online version of this article (10.1186/s12940-019-0465-7) contains supplementary material, which is available to authorized users.

## Background

Fluoridation has been used globally for decades to reduce dental caries. Results of earlier studies [1, 2] have demonstrated that optimally fluoridated water prevents dental caries without posing serious risks to human health. However, a U.S. National Research Council (NRC) review on fluoride toxicity indicated that high fluoride levels in drinking water (≥4 ppm) are associated with increased risk of enamel fluorosis, bone fractures and joint pain [3]. Therefore, the NRC committee recommended that the U.S. Environmental Protection Agency’s (EPA) Maximum Contaminant Level Goal for fluoride (4 ppm) should be lowered.

In addition to the effects of fluoride on bone and teeth, growing evidence is showing potential impact of fluoride exposure on the alterations in reproductive hormones, fertility, and possibly timing of sexual maturity [3]. However, studies of the association between fluoride exposure and sexual maturity are rare. One animal study reported that fluoride exposure can cause earlier sex maturation in female Mongolian gerbils [4]. An ecological study of girls in New York observed an earlier age at menarche in relation to fluoride exposure [5], while another ecological investigation of Hungarian girls reported no associations [6]. On the other hand, more recent studies have related fluoride exposure to decreased sex hormones in humans [7–9].

The existing epidemiological investigations were limited by the ecological design to estimate childhood exposure to fluoride using drinking water concentrations at population level (i.e. lacked individual fluoride biomarkers) and using single indicator of pubertal development (i.e. menarcheal age). In addition, no analyses have evaluated the association between fluoride exposure and sexual maturity in boys. Therefore, the objective of this cross-sectional study was to address these aforementioned knowledge gaps by examining the association between childhood exposure to fluoride levels (via specific-gravity adjusted urinary measures) and physical markers of pubertal development in 157 boys and 176 girls at age 10–17 years residing in Mexico City. Physical markers of pubertal development in girls included pubic hair, breast maturation and age at menarche, and in boys included pubic hair, genital maturation and testicular volume.

## Methods

### Study population

In this study, we included participants from the Early Life Exposures in Mexico to ENvironmental Toxicants (ELEMENT) project, which is an on-going birth cohort study of both pregnant women and their offspring in Mexico City. Child participants (*n* = 554) were invited to participate in the follow-up study between 2015 and 2017 based on the availability of maternal biological samples and age 10–17 years (thus likely to reach different stages of pubertal transition). Pertinent details of ELEMENT study, such as recruitment, eligibility criteria and collection of maternal information have been described previously [10, 11]. During the follow-up visit, child participants provided urine samples, completed anthropometric measures and physician-assessed Tanner stages. Interview-based questionnaires were also completed at the visit. Among these 554 participants, 347 had data on urinary fluoride with measured specific gravity. We further excluded participants who had no measurement of pubertal development, leaving 339 children. Of these 339 participants, 333 had complete information on the covariates of interest including child age and body mass index (BMI) z-score, number of siblings at birth, maternal education and marital status and were included in the final analysis.

Research protocols were approved by the Institutional Review Board at University of Michigan and the University of Indiana, and the Research, Biosafety and Ethics in Research at the Mexico National Institute of Public Health. Prior to enrollment, informed consent from mothers and informed assent from offspring were obtained.

### Urinary fluoride

Spot urine samples of about 5 mL were collected in fluoride-free vials, frozen immediately at the research site, and shipped and stored at − 80 °C at University of Michigan School of Public Health. The samples were analyzed at the Indiana University School of Dentistry Oral Health Research Institute. Urinary fluoride levels were assessed using ion-selective electrode-based diffusion assays following standardized analytic methods. Detailed information regarding the protocol, validation and quality control of measuring fluoride in urine has been described elsewhere [11–13]. Specific gravity was used to correct for variations in urine dilution at the time of measurement and was analyzed using a handheld digital refractometer (Atago Co., Ltd., Tokyo, Japan). Specific gravity-adjusted urinary fluoride concentrations (UF_sg_) were calculated for each participant using the following formula: UF_sg_ = UF_child_ * (1.02–1) / (specific gravity-1). The median level of specific gravity is 1.02 in the study population.

### Pubertal indicators

Tanner staging with a range from stage 1 indicating pre-pubertal to stage 5 indicating fully mature was used to assess pubertal development of all participants [14]. Tanner staging of breast and pubic hair growth in girls as well as Tanner staging of genitalia and pubic hair growth in boys were assessed by a trained pediatrician using standardized protocols*.* The assessment of pubertal stages has been validated by comparing with a panel of serum sex hormones in ELEMENT children [15]. Menarche was measured via a self-reported questionnaire, where girls at age 10–17 years were asked if and when they had initiated menses. In boys, testicular volume was used as an additional indicator of puberty. Testicular volume (right and left) was determined by comparing with a Prader orchidometer (range 1–25 mL). In the present analysis, the larger volume of the right or left testicles was used. Testicular volume ≥ 20 mL was defined as matured stage [14].

### Statistical analysis

Univariate and bivariate analyses were conducted. The distribution of specific gravity-adjusted urinary fluoride was right skewed. Given that normality on independent variable is not required for logistic or Cox proportional-hazard regression models, we did not transform the non-normal variable. We compared the differences in key covariates between included and excluded participants due to missing data. We also compared the characteristics of participating children by sex. We performed t-test, Wilcoxon rank-sum test or the analysis of variance (ANOVA) to assess the differences between continuous variables, and adopted Chi-square test to examine the differences between categorical variables. Covariates considered in this analysis as predictors of puberty or potential confounders included child age and BMI z-scores, maternal education [16] and marital status [17], as well as number of siblings at birth [18]. BMI was calculated and then converted to an age- and sex- specific z-score based on the World Health Organization (WHO) standard curves for children aged from 5 to 19 years [19]. Lead has also been shown to be associated with pubertal development. In fact, we previously reported that prenatal and early childhood exposure to lead were associated with later pubertal development in girls [14]. However, in this study, we did not include peripubertal lead levels at age 10–17 years in the models because we did not observe a significant association between peripubertal lead levels and any markers of pubertal development.

We determined the associations between specific gravity-adjusted urinary fluoride and pubertal stages for the maturation of breast, pubic hair and genitalia using multivariate ordinal regression models. Testicular volume (≥20 mL versus < 20 mL) was examined using logistic regression models. Results for ordinal regression models were reported as the odds of reaching a high stage of puberty (breast, pubic hair or genitalia) versus combined low and middle stages, with a one-interquartile range (IQR) increase in urinary fluoride concentrations. Results for logistic regression models were reported as the odds of having fully matured testicular volume (≥20 mL) versus having juvenile testicular volume (< 20 mL), with a one-IQR increase in urinary fluoride concentrations.

We used survival techniques (time-to-event analysis) to examine the association between fluoride concentration and age at menarche in girls, which adequately account for censored data [20]. Hazards ratios (HRs) and 95% confidence intervals (CIs) were estimated using Cox proportional-hazard regression models, which has been widely used to analyze age at menarche. Time to menarche was based on the self-reported age of menarche (years) or right-censored observations using the age at the interview (i.e. for girls who had not initiated menses). First, we included urinary fluoride concentration as a continuous variable in the models. Second, we divided the urinary fluoride into a categorical variable by tertiles. We defined statistical significance as *p* < 0.05. All statistical analyses were performed using SAS (version 9.4; SAS Institute Inc., Cary, NC, USA).

### Sensitivity analysis

In sensitivity analysis, we examined the adjusted prospective association between prenatal exposure to fluoride during pregnancy and pubertal development in boys and girls. Only a small subset of participants had data on prenatal fluoride, as measured by urinary fluoride in any trimester of pregnancy with the adjustment of creatinine in urine (*n* = 90 for boys and *n* = 111 for girls). The mean of all available creatinine-adjusted urinary fluoride during pregnancy was obtained. In addition, we assessed the adjusted prospective association between childhood exposure to fluoride at age 6–12 years and pubertal development in boys and girls. Similarly, only a small subset of participants had data on childhood exposure fluoride, as measured by urinary fluoride at age 6–12 years with the adjustment of specific gravity in urine (*n* = 66 for boys and *n* = 72 for girls). Detailed information regarding the methods for fluoride analysis has been published elsewhere [11].

## Results

In this study, only child age and maternal education (borderline significant) of included children were significantly different from those who were excluded (Table 1). Children included in the study were younger at the visit (mean 13.9 versus 15.5 years, *p* < 0.0001), and their mothers received higher education (mean 11.1 versus 10.6 years, *p* = 0.05). Most of the participating children’s mothers were married (73.6%). Included children had an average BMI z-score at 0.5 and had on average two siblings, and 47.2% were boys. The overall geometric mean and IQR of urinary fluoride (specific gravity adjusted) in participating children were 0.59 mg/L and 0.31 mg/L, respectively.

**Table 1:** Characteristics of included and excluded participants

	Included (total *N* = 333)		Excluded (total *N* = 221)		
	N	Mean (SD) or %	N	Mean (SD) or %	*p*-value
Demographics					
Child’s age (years)	333	13.9 (2.1)	217	15.5 (1.8)	< 0.0001
Sex (male)	157	47.2%	108	49.8%	0.55
BMI-for-age z-score	333	0.5 (1.4)	212	0.5 (1.0)	0.79
Number of siblings at birth	333	2.0 (1.0)	212	2.0 (1.0)	0.34
Maternal education (years)	333	11.1 (3.0)	212	10.6 (2.8)	0.05
Marital status					
Married	245	73.6%	142	67.6%	0.14
Not married	88	26.4%	68	32.4%	

The distributions of key covariates among participating boys and girls are shown in Table 2. When comparing the characteristics of included participants by sex, we did not find significant differences in any key variables. The concentrations of urinary fluoride did not differ significantly across the categories of these key covariates (Table 3). Girls’ mothers who received education > 12 years had the lowest level of urinary fluoride (geometric mean: 0.51 mg/L); however, the difference was modest and not statistically significant (*p* = 0.08). Table 4 shows the distribution of physician-assessed pubertal development of boys and girls at age 10–17 years by physical indicators. One hundred and thirty-seven girls (78.3%) had initiated menarche with mean (SD) menarcheal age at 11.4 years (SD = 1.2).

**Table 2:** Distribution of key covariates among 157 boys and 176 girls

Variables		Min	10%	25%	50%	75%	90%	Max	p-value^a^
Child’s age (years)	Boys	10.7	11.5	12.2	13.4	15.8	17.2	17.7	0.70
	Girls	10.8	11.4	12.0	13.5	15.8	16.8	17.8	
BMI-for-age z-score	Boys	−3.8	−1.6	−0.6	0.5	1.6	2.3	3.5	0.22
	Girls	−2.9	−0.9	−0.3	0.5	1.5	2.2	3.3	
Maternal education (years)	Boys	6.0	9.0	9.0	12.0	12.0	16.0	20.0	0.43
	Girls	2.0	7.0	9.0	11.0	12.0	16.0	21.0	
Number of siblings at birth	Boys	1.0	1.0	1.0	2.0	3.0	3.0	6.0	0.49
	Girls	0.0	1.0	1.0	2.0	3.0	3.0	5.0	
Urinary fluoride (mg/L)^b^	Boys	0.15	0.38	0.48	0.63	0.77	0.97	3.01	0.64
	Girls	0.05	0.34	0.43	0.57	0.75	1.09	2.61	
		Boys			Girls				0.39
Marital status		N	%		N	%			
Married		119	75.8%		126	71.6%			
Not married		38	24.2%		50	28.4%			

^a^Analysis of differences in key covariates by child sex ^b^Specific gravity-adjusted childhood urinary fluoride

**Table 3:** Urinary fluoride levels (mg/L) according to key covariates by sex^a^

Variable		N	Geometric mean (95% CI)	p-value
Boys				
Age (years)	≥12	123	0.61 (0.57, 0.66)	0.35
	< 12	34	0.58 (0.53, 0.65)	
Number of siblings at birth	< 2	55	0.58 (0.53, 0.64)	0.42
	≥2	102	0.62 (0.57, 0.67)	
Maternal Education (years)	< 12	76	0.59 (0.54, 0.65)	0.22
	12	56	0.65 (0.57, 0.73)	
	> 12	25	0.56 (0.48, 0.64)	
Marital status	Married	119	0.62 (0.57, 0.67)	0.10
	Other	38	0.56 (0.50, 0.62)	
Girls				
Age (years)	≥12	127	0.59 (0.53, 0.64)	0.55
	< 12	49	0.55 (0.49, 0.61)	
Number of siblings at birth	< 2	69	0.57 (0.52, 0.63)	0.84
	≥2	107	0.57 (0.52, 0.63)	
Maternal Education (years)	< 12	90	0.57 (0.51, 0.63)	0.08
	12	56	0.63 (0.55, 0.71)	
	> 12	30	0.51 (0.46, 0.56)	
Marital status	Married	126	0.57 (0.52, 0.62)	0.68
	Other	50	0.58 (0.51, 0.67)	

*CI* confidence interval

^a^Specific gravity-adjusted childhood urinary fluoride

**Table 4:** Distribution of physician-assessed secondary sex characteristics^a^

Measure	Stage	N (%)
Boys		
Pubic hair	1	42 (26.8)
	2	23 (14.7)
	3	41 (26.1)
	4	22 (14.0)
	5	29 (18.5)
Genitalia	1	9 (5.7)
	2	30 (19.1)
	3	32 (20.4)
	4	57 (36.3)
	5	29 (18.5)
Testicular volume	Yes (> = 20 ml)	101 (64.3)
	No	56 (35.7)
Girls		
Pubic hair	1	16 (9.4)
	2	53 (31.0)
	3	44 (22.5)
	4	37 (21.6)
	5	21 (12.3)
Breast	1	10 (5.9)
	2	26 (15.2)
	3	49 (28.7)
	4	59 (34.5)
	5	27 (15.8)
Menarche	Yes	137 (78.3)
	No	38 (21.7)

^a^The sample size is 157 for each indicator of puberty in boys. For girls, 171 of 176 girls had available data on pubic hair and breast development, and 175 girls had data on menarche

In boys, we found significant negative associations between specific gravity-adjusted urinary fluoride concentrations and pubic hair growth and genital maturation, controlling for child age and BMI z-score, number of siblings at birth, maternal education and marital status (Table 5). With a one-IQR increase (0.31 mg/L) in urinary fluoride, the odds of reaching a high stage of pubic hair growth decreased by 29% (Odds ratio (OR): 0.71, 95% confidence interval (CI): 0.51–0.98, *p* = 0.03); the odds of reaching a high stage of genitalia decreased by 29% (OR: 0.71, 95% CI: 0.53–0.95, *p* = 0.02) in boys. We did not observe a significant difference in the odds of having matured testicular volume (> = 20 ml) in relation to urinary fluoride levels, although the direction was consistent (OR: 0.90, 95% CI: 0.65–1.27, *p* = 0.56).

**Table 5:** Adjusted odds ratio (95% confidence interval) of physician-assessed pubertal development per IQR (0.31 mg/L) increase in urinary fluoride concentrations in children^a^

	Specific-gravity adjusted childhood urinary fluoride		
	N	OR (95% CI)	p-value
Boys			
Pubic hair	157	0.71 (0.51, 0.98)	0.03
Genitalia	157	0.71 (0.53, 0.95)	0.02
Testicular volume	157	0.90 (0.65, 1.27)	0.56
Girls			
Pubic hair	171	0.86 (0.65, 1.14)	0.29
Breast	171	0.85 (0.63, 1.14)	0.30

*OR* odds ratio, *CI* confidence interval

^a^For pubic hair, genitalia and breast, all estimates are from ordinal regression models. For testicular volume, all estimates are from logistic regression models. All models adjusted for child age and BMI z-score, number of siblings at birth, maternal education and marital status

In girls, we found that specific gravity-adjusted urinary fluoride was negatively associated with pubertal stages for pubic hair growth and breast maturation adjusting for the same covariates, although the associations did not reach significance (pubic hair OR: 0.86, *p* = 0.29; breast OR: 0.85, *p* = 0.30) (Table 6). Similarly, urinary fluoride was not significantly related to menarcheal age in girls regardless of treating fluoride as a continuous or a categorical variable, adjusted for number of siblings at birth, maternal education and marital status, as well as child BMI z-score. However, the direction of these associations was negative.

**Table 6:** Adjusted hazard ratio (95% confidence interval) of self-reported menarche according to urinary fluoride in girls^a^

	Specific-gravity adjusted childhood urinary fluoride		
	N	HR (95% CI)	p-value
Menarche			
Continuous fluoride (mg/L)	175	0.82 (0.51, 1.33)	0.42
1st tertile (< 0.48)	68	Reference	–
2nd tertile (0.49–0.69)	53	0.98 (0.64, 1.51)	0.94
3rd tertile (0.70–2.61)	55	0.76 (0.50, 1.17)	0.21

*HR* hazard ratio, *CI* confidence interval

^a^All estimates are from Cox proportional-hazard models adjusted for maternal education and marital status, child BMI z-score and number of siblings at birth

In sensitivity analysis, we examined the adjusted prospective association of creatinine-adjusted urinary fluoride during pregnancy (See Additional file 1: Table S1 and S2) and specific gravity-adjusted childhood urinary fluoride (age 6–12 years) (See Additional file 1: Table S3 and S4) with physical markers of puberty in a much smaller subset of our participants who had available data. Overall, we found no significant associations with any measures of pubertal development in these boys or girls, although girls with childhood urinary fluoride at age 6–12 years in the 3rd tertile had a later age at menarche compared with girls in the 1st tertile (Hazard ratio (HR): 0.37, 95% CI: 0.16–0.86, *p* = 0.02) (See Additional file 1: Table S4).

## Discussion

This is the first study that examines the relationship between a biomarker of fluoride exposure and multiple physical indicators of pubertal development in children of both sexes. Our findings suggest that fluoride exposure at the levels observed in this study during peripubescence is associated with later pubertal development for pubic hair growth and genital maturation in Mexican boys. We found a negative association between peripubertal fluoride and pubertal development in girls, but the results did not reach significance.

An ecological study of 804 Hungarian girls at age 10–19 years observed no substantial differences in the age at menarche between girls living in the high-fluoride town Kunszentmárton (with life-long exposure to fluoridated drinking water at 1.09 mg/L) and girls living in the low-fluoride town Kiskunmajsa (0.17 mg/L) [6]. On the contrary, an animal study of 24 female Mongolian gerbils revealed earlier sex maturation (i.e. earlier vaginal opening and the development of ventral gland) occurred in high fluoride treatment group (3.7 mg/kg/day) versus low fluoride treatment group (0.7 mg/kg/day) [4]. An earlier ecological study of 405 girls aged 7–18 years who had been exposed to fluoridated water up to 10 years showed that the average age at menarche was 12 years among girls in fluoridated Newburgh, New York (fluoridated drinking water 1.2 mg/L), versus 12 years 5 months among girls in Kingston, where water was not fluoridated (essentially fluoride-free) [5]. The main fluoride exposure sources of the published ecological studies were different from ours: their participants received fluoride through water supply, while our participants were exposed to fluoride through table salt at 200–250 mg/kg salt. In addition to the ecological design, these studies are limited by reliance on self-reported age at menarche, which can be susceptible to recall bias. It has been proposed by Luke that earlier pubertal onset may be a result of reduced release of melatonin from pineal gland in response to fluoride exposure [4]. Decreased melatonin has been shown to increase gonadotropins and subsequently stimulate sex hormones and eventually accelerate pubertal development [21], but the effect of fluoride on the melatonin production has not been well characterized. A more recent animal study provides some evidence for lower estradiol and progesterone in serum, and damaged ovaries in female rats receiving high fluoride treatment (100 mg/L) [22], which may lead to a delayed pubertal development. More studies in this area are warranted.

In males, previous investigations have linked fluoride exposure to decreased sex hormones in humans. A Mexican study revealed that male adults with an average urinary fluoride at 3.2 mg F/g creatinine had lower serum testosterone and inhibin-B levels compared to those with an average urinary fluoride at 1.6 mg F/g creatinine [7]. Similarly, the lower concentration of testosterone in serum was observed in male adults who had high versus low urinary fluoride (mean 2.64 versus 0.94 mg/L) in Henan, China [9]. An Indian analysis also found that male adult patients with skeletal fluorosis (mean urinary fluoride 3.5 mg/L) had lower concentration of testosterone compared with those who were healthy [8]. Although decreased melatonin levels due to fluoride exposure could potentially increase testosterone as described above [4], it has been suggested that fluoride may directly impair the structure and function of Leydig cells and disrupt the activities of the hypothalamic–pituitary–thyroid (HPT) axis, and hence the release of testosterone can be reduced [23, 24]. In addition, Luke’s study showed that the testicular weight in gerbils at 16 weeks was lower in high fluoride treatment group versus low fluoride treatment group [4]. It is possible that the decreased levels of testosterone and inhibin-B may in turn postpone puberty, which may explain the negative associations observed in our study.

According to a recent review [25], late puberty has been associated with low self-esteem, disruptive behavior disorder and substance use in young adults, depression and anxiety in later adulthood. Late puberty may also be associated with an increased risk of bone fracture and coronary heart disease in adults [25–27].

Unlike our study, the majority of previous studies that measured urinary fluoride concentrations did not correct for variations in urine (e.g. adjust for urinary specific gravity levels). A study of 294 Canadian children aged 3–12 years reported a mean specific gravity-adjusted urinary fluoride concentration at 0.80 mg/L [28]. Seventy-eight children from Iran at age 4 years had a mean value of urinary fluoride concentration (specific gravity adjusted) at 0.73 mg/L [29]. A Mexican study of 108 children at age 6–12 years residing in rural San Luis Potosi reported a mean specific gravity-adjusted urinary fluoride level at 3.14 mg/L [30]. Here we compared the urinary fluoride levels in our cohort (geometric mean: 0.59 mg/L) with the levels reported by other populations, although current studies were quite limited.

The study has some limitations. The sample size of our analysis is small for modeling ordinal outcomes, which may limit the power to detect significant associations between fluoride exposure and pubertal development. The use of spot urine samples (versus 24 h collections) also provides an additional limitation, since fluoride concentration values have been reported to fluctuate at different collection points [31]. As noted above, the use of self-reported age at menarche can be subject to recall bias. However, previous studies have shown that the actual menarcheal age was highly correlated with self-reported menarcheal age within 5 years of follow-up among peripubertal girls [32], within 7 years [33] among women and up to 33 years starting at age 7–9 years [34]. This study also has several advantages over the existing human studies including the use of individual biomarkers of fluoride exposure and multiple markers of physician-assessed pubertal development in both boys and girls.

## Conclusions

We found a significant association between an increase in peripubertal fluoride exposure at the level observed in our study population and later pubertal development in Mexican boys at age 10–17 years. However, pubertal development in girls was not significantly associated with fluoride exposure, which is inconsistent with previous animal and ecological studies. Further research with a larger sample size and multiple collections of urine samples is needed to confirm our findings.

## Additional file

Additional file 1: **Table S1.** Adjusted odds ratio (95% confidence interval) of physician-assessed pubertal development in children aged 10–17 years per IQR (0.54 mg/L) increase in prenatal urinary fluoride concentrations. **Table S2.** Adjusted hazard ratio (95% confidence interval) of self-reported menarche in girls aged 10–17 years according to prenatal urinary fluoride. **Table S3.** Adjusted odds ratio (95% confidence interval) of physician-assessed pubertal development in children aged 10–17 years per IQR (0.59 mg/L) increase in childhood urinary fluoride concentrations (at age 6–12 years). **Table S4.** Adjusted hazard ratio (95% confidence interval) of self-reported menarche in girls aged 10–17 years according to childhood urinary fluoride (at age 6–12 years). (DOCX 25 kb)

## Abbreviations

ANOVA: The analysis of variance
BMI: Body mass index
CI: Confidence interval
ELEMENT: Early Life Exposures in Mexico to ENvironmental Toxicants
EPA: Environmental Protection Agency
HPT axis: The hypothalamic–pituitary–thyroid axis
IQR: Interquartile range
OR: Odds ratio
U.S NRC: U.S. National Research Council
WHO: World Health Organization
