# Supplementary material for: Fluoride exposure and pubertal development in children living in Mexico City
Source: Environ Health. 2019 Mar 29;18:26. doi: 10.1186/s12940-019-0465-7 (PMC6439980; doi:10.1186/s12940-019-0465-7)
Supplement: Supplementary file 1 — Table S1. Adjusted odds ratio (95% confidence interval) of physician-assessed pubertal development in children aged 10–17 years per IQR (0.54 mg/L) increase in prenatal urinary fluoride concentrations. Table S2. Adjusted hazard ratio (95% confidence interval) of self-reported menarche in girls aged 10–17 years according to prenatal urinary fluoride. Table S3. Adjusted odds ratio (95% confidence interval) of physician-assessed pubertal development in children aged 10–17 years per IQR (0.59 mg/L) increase in childhood urinary fluoride concentrations (at age 6–12 years). Table S4. Adjusted hazard ratio (95% confidence interval) of self-reported menarche in girls aged 10–17 years according to childhood urinary fluoride (at age 6–12 years). (DOCX 25 kb) [file 12940_2019_465_MOESM1_ESM.docx]

Table S1. Adjusted odds ratio (95% confidence interval) of physician-assessed pubertal development in children aged 10-17 years per IQR (0.54 mg/L) increase in prenatal urinary fluoride concentrations^a^

|  | Creatinine-adjusted prenatal urinary fluoride | | | |
| --- | --- | --- | --- | --- |
|  | N | OR (95% CI) | | p - value |
| Boys |  |  | |  |
| Pubic hair | 90 | 1.33 (0.88, 2.01) | | 0.18 |
| Genitalia | 90 | 0.83 (0.56, 1.23) | | 0.35 |
| Testicular volume | 90 | 1.01 (0.63, 1.63) | | 0.96 |
|  |  |  | |  |
| Girls |  |  |  |  |
| Pubic hair | 111 | 0.95 (0.56, 1.62) | | 0.86 |
| Breast | 111 | 1.13 (0.66, 1.92) | | 0.65 |

Note: OR, odds ratio; CI, confidence interval. ^a^For pubic hair, genitalia and breast, all estimates are from ordinal regression models. For testicular volume, all estimates are from logistic regression models. All models adjusted for child age and BMI z-score, number of siblings at birth, maternal education and marital status.

Table S2. Adjusted hazard ratio (95% confidence interval) of self-reported menarche in girls aged 10-17 years according to prenatal urinary fluoride^a^

|  | Creatinine-adjusted prenatal urinary fluoride | | |
| --- | --- | --- | --- |
|  | N | HR (95% CI) | p - value |
| Menarche |  |  |  |
| Continuous fluoride (mg/L) | 115 | 0.87 (0.47, 1.61) | 0.66 |
| 1^st^ tertile (<0.70) | 41 | Reference | - |
| 2^nd^ tertile (0.73-1.05) | 47 | 0.82 (0.50, 1.37) | 0.45 |
| 3^rd^ tertile (1.12-2.39) | 27 | 0.74 (0.40, 1.36) | 0.33 |

Note: HR, hazard ratio; CI, confidence interval. ^a^All estimates are from Cox proportional-hazard models adjusted for maternal education and marital status, child BMI z-score and number of siblings at birth.

Table S3. Adjusted odds ratio (95% confidence interval) of physician-assessed pubertal development in children aged 10-17 years per IQR (0.57 mg/L) increase in childhood urinary fluoride concentrations (age 6-12 years)^a^

|  | Specific gravity-adjusted childhood urinary fluoride | | | |
| --- | --- | --- | --- | --- |
|  | N | OR (95% CI) | | p - value |
| Boys |  |  | |  |
| Pubic hair | 66 | 1.27 (0.54, 3.01) | | 0.56 |
| Genitalia | 66 | 0.99 (0.62, 1.58) | | 0.96 |
| Testicular volume | 66 | 1.10 (0.64, 1.87) | | 0.74 |
|  |  |  | |  |
| Girls |  |  |  |  |
| Pubic hair | 72 | 0.82 (0.55, 1.23) | | 0.34 |
| Breast | 72 | 0.80 (0.54, 1.20) | | 0.28 |

Note: OR, odds ratio; CI, confidence interval. ^a^For pubic hair, genitalia and breast, all estimates are from ordinal regression models. For testicular volume, all estimates are from logistic regression models. All models adjusted for child age and BMI z-score, number of siblings at birth, maternal education and marital status.

Table S4. Adjusted hazard ratio (95% confidence interval) of self-reported menarche in girls aged 10-17 years according to childhood urinary fluoride concentrations (age 6-12 years)^a^

|  | Specific gravity-adjusted childhood urinary fluoride | | |
| --- | --- | --- | --- |
|  | N | HR (95% CI) | p - value |
| Menarche |  |  |  |
| Continuous fluoride (mg/L) | 76 | 0.69 (0.38, 1.24) | 0.22 |
| 1^st^ tertile (<0.45) | 23 | Reference | - |
| 2^nd^ tertile (0.46-0.81) | 28 | 0.67 (0.35, 1.31) | 0.25 |
| 3^rd^ tertile (0.93-3.46) | 25 | 0.37 (0.16, 0.86) | 0.02 |

Note: HR, hazard ratio; CI, confidence interval. ^a^All estimates are from Cox proportional-hazard models adjusted for maternal education and marital status, child BMI z-score and number of siblings at birth.
